# Building a safety buffer for European food security: the role of small-scale food production and local ecological and gastronomic knowledge in light of COVID-19

**DOI:** 10.12688/openreseurope.13138.2

**Published:** 2021-10-21

**Authors:** Renata Sõukand, Raivo Kalle, Michele Filippo Fontefrancesco, Andrea Pieroni

**Affiliations:** 1 DAIS, Ca’ Foscari University of Venice, Mestre, 30172, Italy; 2 University of Gastronomic Sciences, Pollenzo, 12042, Italy

**Keywords:** food security, traditional ecological knowledge, Europe, COVID-19, local production, foraging, education

## Abstract

The lockdown caused by the coronavirus 2019 disease (COVID-19) has created a situation in which food availability is affected not only by the availability of money but also by the availability of food itself. On the basis of five pillars, including 1) supporting community-based farming, 2) defending small firms, 3) developing narratives on the high value of local food, 4) encouraging subsistence gardening and foraging in the wild, and 5) promoting local ecological and gastronomic knowledge, the essay points a way forward to attain greater sustainability and resilience of safe food chains that starts with reassessing the relevance of local food systems.

## Restarting from the local level

Coronavirus disease 2019 (COVID-19) has pointed out a crucial weakness of European food security. Since their early implementation, European agricultural policies have dealt with issues of food security. However, they have focused mainly on achieving food security on an international level, within the European community, while overlooking the importance of local and regional levels; indeed, in case of a food shortage in a specific region, everything could be procured within the broader space of the European market. This strategy has deeply affected the organization of agricultural practices on farms, pushing agriculture toward production intensification and specialization ( Emery *et al.,* 2017; Stock *et al.,* 2014). It has also changed the role of traditional food practices. In particular, locally foraged food has been turning into haute-cuisine ( Łuczaj *et al.,* 2012) and small-scale production or subsistence gardening into a niche activity accessible to the few enthusiasts – visible, yet marginal. The lockdown caused by COVID-19 has created a situation unprecedented since WWII, in which food availability is affected not only by the availability of money but also by the availability of food itself.

Closed borders have caused delays in food supplies coming from remote regions, and also the inability to produce locally grown food in systems based on a foreign workforce (see also Nori & Farinella, 2020). In the worldwide media, there have been numerous reports on failures to collect vegetables from cultivated fields in spring 2020 (e.g., asparagus in Germany, or tomatoes in Italy). At the same time, other springtime tasks, such as land preparation and planting, were restricted due to the shortage of field workers, of whom a significant proportion are not local. In Italy, for example, farmer associations pointed out the scarcity of farm laborers that were required for the completion of the harvest, while in Estonia, dairy farm owners complained that Ukrainian workers, who were not able to return from spring vacation, are irreplaceable, as they have the requisite skills and will work for considerably lower wages compared to locals.

The circumstances we are experiencing call for a reimagining of agricultural policies. In this process, that necessitates the contribution of professionals, policymakers, researchers and civil society, we want to highlight several factors that must be considered for the mitigation of risks related to food availability in times of crisis. Leaving aside the issue of food inequality, which would open discussion on the consumer dimension not addressed in this essay, we propose a set of five safety pillars which could serve as a temporary buffer for turbulent times:

1) Supporting community-based farming.

2) Defending small firms.

3) Developing narratives on the high value of local food.

4) Encouraging subsistence gardening and foraging in the wild.

5) Promoting local ecological and gastronomic knowledge.

## Supporting community-based farming

The first aspect is closely related to the “small is beautiful” concept defined by British economist Ernst Friedrich Schumacher (1911–1977) in the 1970s ( Schumacher, 2011). The need for workforce recruitment primarily relates to large-scale farming and animal husbandry. Heavily relying on the work of people external to the local community ( D'souza & Ikerd, 1996), the past few months have shown that these farms are not sustainable in the event of being cut-off from external resources. Farms which operate using locally available resources, including the workforce, are potentially more sustainable ( D'souza & Ikerd, 1996) and can withstand the consequences of a lockdown or any other similar disturbance in food availability. Jouzi *et al.* (2017) stated: "Consequently, poor farmers can increase their yield by applying OF [organic farming] practices which are mostly based on agroecological principles". However, small (organic) farms are less demanding on the environment and yield proportionally more profit than large ones relying on subsidies, as shown in a recent study (before Brexit) in the UK ( Clark *et al.,* 2019), which is predominantly the land of corporative farming, and so this applies not only to “poor farmers”.

While the smaller internal buffer space and the higher cost of sustainable production may make such farms vulnerable in the market, support could be provided by governmental subsidies dedicated specifically to small-scale food chains and “food communities”. The importance of small farms and producers in food security is also highlighted in the European Green Deal's "Farm to Fork strategy: for a fair, healthy and environmentally friendly food system" ( European Green Deal).

## Defending small firms

The form of agriculture we advocate resisted centralization and is a network economy based on networks of small firms, which should be sustained. Small enterprises are vital to creating a competitive, flexible and resilient form of production, with strong local positive impacts on local communities ( Piore & Sabel, 1984). However, small firms are not resilient to major economic crises due to their simple business structure and lack of capitalization. COVID-19 has heavily impacted these enterprises ( Bartik *et al.,* 2020). Small farms are experiencing similar difficulties and there is uncertainty in other sectors as well, such as tourism and catering. In this context, neoliberalist laissez-faire is detrimental. Within a framework of a broader catastrophe recovery plan ( Baldwin & Weder di Mauro, 2020), governments on a national and an international level should support small and medium-sized enterprises, especially in the strategic sector of food production, to preserve this fundamental element of European economy and progress toward the goal of sustainable and responsible production.

## Developing narratives on the high value of local food

Assistance should not come just in terms of monetary support, but also in the form of support for the reputation of local food production. New EU member states have come from the former socialist bloc, which destroyed small-scale farms and caused severe humanitarian issues by introducing collective farming and (in the Soviet Union) the subsequent deportation of those refusing to participate. This created a discontinuity in local voluntary co-production in village communities and the loss of small-scale operations, and also destroyed the narrative on the importance of local foods in those regions.

Following the example of the Slow Food movement, which was created in Italy, as well as the New Nordic Cuisine movement in Northern Europe, the idea of local food has been gradually introduced in post-socialist countries, but it needs more involvement for wider acceptance. In the face of this particular situation, supporting small-scale farms in the launch of community-supported agricultural platforms is crucial, as is the implementation of educational policies aimed at promoting the responsibility of the individual, making food purchasing decisions on the basis of their sustainability.

## Encouraging subsistence gardening and foraging in the wild

To strengthen food security, Europe should look back to the importance of the practices of subsistence gardening and foraging. The need for self-sufficiency in Eastern Europe ( Gudeman & Hann, 2015) resulted in the persistence of subsistence gardening among the general population (every family had a piece of land on which to grow their vegetables, as the retail system was not able to secure a sufficient food supply) for a much longer period than the rest of Europe. After the collapse of the Soviet Union at the beginning of the 1990s, the economic situation worsened drastically but, in Ukraine for example, only a few people starved to death as there were other economic practices that allowed people to survive in times of crisis ( Round *et al.,* 2010). The investment in these practices provided widespread family-scale subsistence food production and an informal market that allowed for the exchange of self-grown products ( Sõukand *et al.,* 2020). Despite being deeply rooted in the acute need for food during and after the communist period, allotment gardening in Eastern Europe has become more pleasure-oriented ( Klepacki & Kujawska, 2018) and is currently considered economically impractical ( Sowińska-Świerkosz *et al.*, 2021).

Modern urban foraging is also frequently motivated by personal enjoyment ( Landor-Yamagata *et al.*, 2018). The knowledge is currently held by a limited number of enthusiasts and/or professionals ( Łuczaj *et al.*, 2021) who were well mobilized through social media during the Covid-19 lock-downs ( Townsend, 2020), yet such practices are not widespread among the general, rather biophobic, population.

Foraging (wild food plant collection) trajectories in Europe have historically been mainly twofold: one has concerned synanthropic plants and assimilable plants and the other has focused on non-timber forest products (especially wild fruits and mushrooms). The former kind of foraging has been practiced since the Neolithic Revolution, and then especially, but not exclusively, in the Mediterranean and Near East. It has been centered on synanthropic environments and plant species, and these practices were and continue to be primarily managed by women ( Pieroni *et al.*, 2018).

A male kind of post-Neolithic foraging also emerged, however, but this was centred on thistles and managed by male shepherds ( Mattalia *et al.*, 2020). The wild plant ingredients of this foraging approach have frequently been introduced in the domestic economy circuit and have been treated similar to products from private home gardens; moreover, they were often traded in the markets by women. 

More interestingly, the ingredients of the second kind of foraging (which did not emerge from plant farming or animal breeding) has been more important in Middle and Northern Europe and these activities have mainly taken place in communal environments, i.e., forests. In many cases, the foraged species became the object of very sophisticated social negotiations, i.e., within the social institutions of gift giving and goods exchange, and marketed only when the work done to gather the plant was significant and/or the perceived value of the collected plants was high. The products of the second kind of foraging have been more important for fostering community cohesion and social sustainability.

The above mentioned clearly shows that foraging has not only represented a way of procuring food, but also an activity well integrated into local socio-ecological systems and should be fostered at different levels within the framework of implementing healthy sustainable food systems.

From this perspective, the importance of educational and community programs regarding school and community gardens launched across the continent in the past few decades is apparent. These initiatives are important to locally integrate the food cycle (e.g., promoting the local production of fruits and vegetables in a school that are then processed by the school canteen) and to re-embed food knowledge among members of the community.

While we do not advocate the complete self-sustainability of households or the return to widespread foraging as a means of survival, we do encourage the involvement of a considerable proportion of citizens in subsistence gardening practices as well as the teaching of foraging techniques (including recognition in the wild of edible plants and their preparation as food) at the school level. While the generations that were once able to care for food gardens or forage in the wild are still alive, the embodied knowledge is “still there” and should therefore be transmitted in embodied form.

## Promoting local environmental and gastronomic knowledge

Evolutionally, food security has relied upon and has been secured by food, which is organically part of the local environment, often “coalesced” into specific food biocultural refugia ( Barthel *et al.,* 2013). Collecting it requires a certain set of skills, starting with knowledge of the habitats of wild ingredients, recognition, gathering/hunting times, the preparation involved in obtaining a diversity of dishes, and assembling them within given aesthetics; this is local environmental and gastronomic knowledge. While wild food alone cannot possibly provide all the caloric and nutritive needs of large groups of people or for long periods, it can serve as a reservoir in situations of extreme need. Thus, recognizing the environmental resources to be used as food and the techniques needed to make them edible, in other words, traditional gastronomic knowledge, is evolutionally the most important pillar of food security ( Berkes, 1999; Pieroni *et al.,* 2016).

Traditional gastronomic knowledge is embedded in the local space as it develops over generations and reflects the conditions and customs of a specific socio-ecological space. Such embedded knowledge is highly coevolved and transformative, but also quite vulnerable in times of dominating industrial productions and other globalisation-centred processes. While these processes that reshaped the planet over the past century have largely eroded traditional gastronomic knowledge, fostering the complex and inextricable link between biodiversity and cultural customs related to food and gastronomy can contribute in a variety of ways to the well-being of humans and their *Oikos*, particularly in this moment of crisis. Thus, the study of the complex interactions between human societies, food, and their environment – what has been defined as *gastronomic ethnobiology* – is a crucial pillar for fostering food security, food sustainability, and especially community-centred food sovereignty.

## The way forward

The destruction of food systems caused by COVID-19 still seems mild, more like an “exercise”, or a strong warning before the results of global change, including massive biodiversity loss, reveal themselves on an everyday level. It has shown us that basic skills related to the production of food, as a primary necessity for human life, should be more evenly distributed within society to assure people that they will be able to provide for themselves even if centralized supplies cease to exist. While still enjoying the unstable benefits of global food exchange and intensive food production, all European countries should not only safeguard the sustainability of local food production but also nurture small-scale producers, subsistence farming and local/traditional ecological and gastronomic knowledge in particular, as these contribute to a natural buffer in turbulent times. In this framework, we need to re-write the narrative of feeding the hungry and shift focus to the importance of locally produced/gathered food and the ability of humans to feed themselves by securing the proper means of doing so.

In this context, we suggest that positive actions should be directed towards increasing the flexibility of existing food-systems:

1) Finding better ways to support small-scale farming and the development of close-to-home food chains.
2) Promoting subsistence gardening/permaculture as a lifestyle in both rural and urban settings, securing the availability of productive land or other designated spaces for everyone who is willing to continue or learn food cultivation or livestock rearing for their own family’s needs.
3) Supporting community-centred work and equipment sharing to facilitate the involvement of new technologies in local food production.
4) Revising current legislation to support foraged/self-grown/home-prepared food production and facilitate access to the market with less severe regulations compared with industrially made food.
5) Documentation of the local uses of and culinary art linked to widely available wild food ingredients in order to prepare culturally sensitive education platforms.
6) Incorporating into education programs practical exercises embodying the use of local ecological knowledge, including foraging for wild food plants, cultivating vegetables in school gardens, and occasionally visiting small-scale farms so that town-born children also understand where food comes from and what to do to obtain it.

## Data availability

No data are associated with this article.
