# Supplementary material for: Building a safety buffer for European food security: the role of small-scale food production and local ecological and gastronomic knowledge in light of COVID-19
Source: Open Res Eur. 2021 Oct 21;1:10. Originally published 2021 Mar 24. [Version 2] doi: 10.12688/openreseurope.13138.2 (PMC10445880; doi:10.12688/openreseurope.13138.2)
Supplement: Peer review history

# Peer review: Reviewer response for version 2

**DOI:** 10.21956/openreseurope.15326.r27888

**Published:** 2021-10-28

**Authors:** Łukasz Łuczaj

**Affiliations:** 1 Institute of Biology and Biotechnology, University of Rzeszów, Rzeszów, Poland

The article improved and is nearly in its final form.

 Only the sentence "A male kind of post-Neolithic foraging also emerged, however, but this was centred on thistles and managed by male shepherds ( Mattalia *et al.*, 2020)." may be unclear for readers. I think authors are using some rare specific example and are not even using Latin names of "thistles". Either remove this sentence, shorten it or explain better.

Is the argument persuasive and supported by appropriate evidence?

Partly

Is the work clearly and cogently presented?

Yes

Is the topic of the essay discussed accurately in the context of the current literature?

No

Does the essay contribute to the cultural, historical, social understanding of the field?

Yes

Reviewer Expertise:

Foraging, traditional food gathering, wildlife gardening, rewilding.

I confirm that I have read this submission and believe that I have an appropriate level of expertise to confirm that it is of an acceptable scientific standard.

# Peer review: Reviewer response for version 2

**DOI:** 10.21956/openreseurope.15326.r27889

**Published:** 2021-10-25

**Authors:** Mark Nesbitt

**Affiliations:** 1 Royal Botanic Gardens, Kew, Richmond, UK

Thank your for your response. I'm sure this paper will prompt further discussion on the place of local initiatives in food systems.

Is the argument persuasive and supported by appropriate evidence?

Partly

Is the work clearly and cogently presented?

Yes

Is the topic of the essay discussed accurately in the context of the current literature?

Partly

Does the essay contribute to the cultural, historical, social understanding of the field?

Yes

Reviewer Expertise:

Good general knowledge of ethnobotany

I confirm that I have read this submission and believe that I have an appropriate level of expertise to confirm that it is of an acceptable scientific standard.

# Peer review: Reviewer response for version 1

**DOI:** 10.21956/openreseurope.14208.r27136

**Published:** 2021-08-20

**Authors:** Mark Nesbitt

**Affiliations:** 1 Royal Botanic Gardens, Kew, Richmond, UK

This is a timely response to the recent COVID crisis (supplemented by the effects of Brexit in the UK), which have demonstrated the fragility of national and international networks of food production and supply.

 The cultural importance of local, small-scale food production and consumption is well-demonstrated, drawing on ethnobotanical literature and experience of current projects such as Slow Food. However, to be fully persuasive, two more aspects could be addressed.

 The first is the economic framework of small-scale farming and food preparation. Globally, economies have moved away from industry-specific subsidies - at least those which are visible. How could existing mechanisms in Europe be repurposed to provide this support? A successful example is the reorientation of agricultural subsidies to support biodiversity conservation - could that be extended?

 Second is the question of equality of access. It is likely that food from small-scale production will be substantially more expensive (in a European context where labour is costly) than from large-scale. Foraging and community gardens are unlikely to be accessible to, say, residents of deprived urban areas. In any case, they would not have the time free for these activities. There is a significant literature on food inequality in urban areas that would be worth exploring. 

 Researchers have already noticed "the contemporary food sector...bifurcating into two main ‘zones’ of production: standardized, specialized production processes responding to economic standards of efficiency and competitiveness on the one hand; localized, specialized production processes attempting to trade on the basis of environmental, nutritional, or health qualities on the other." (Sonnino & Marsden). There seems a high risk that affluent consumers could enjoy the cash or time-expensive products advocated here, while leaving behind significant sectors of society.

 See the articles below and a significant wider literature on critical food geographies and food networks that is relevant to this subject.

 Dixon, J., Omwega, A. M., Friel, S., Burns, C., Donati, K., & Carlisle, R. (2007). The health equity dimensions of urban food systems. *Journal of Urban Health*, *84*(1), 118-129.

 Sonnino, Roberta, and Terry Marsden. "Beyond the divide: rethinking relationships between alternative and conventional food networks in Europe." *Journal of economic geography* 6.2 (2006): 181-199.

Is the argument persuasive and supported by appropriate evidence?

Partly

Is the work clearly and cogently presented?

Yes

Is the topic of the essay discussed accurately in the context of the current literature?

Partly

Does the essay contribute to the cultural, historical, social understanding of the field?

Yes

Reviewer Expertise:

Good general knowledge of ethnobotany

I confirm that I have read this submission and believe that I have an appropriate level of expertise to confirm that it is of an acceptable scientific standard, however I have significant reservations, as outlined above.

## References

[1] : The health equity dimensions of urban food systems. *J Urban Health* .2007;84(3 Suppl) : 10.1007/s11524-007-9176-4 i118-29 10.1007/s11524-007-9176-4 17401697PMC1891642

[2] : Beyond the divide: rethinking relationships between alternative and conventional food networks in Europe. *Journal of Economic Geography* .2006;6(2) : 10.1093/jeg/lbi006 181-199 10.1093/jeg/lbi006

# Response

**Published:** 2021-10-16

**Authors:** Raivo Kalle

**Affiliations:** University of Gastronomic Sciences, Italy

*However, to be fully persuasive, two more aspects could be addressed. The first is the economic framework of small-scale farming and food preparation. Globally, economies have moved away from industry-specific subsidies - at least those which are visible. How could existing mechanisms in Europe be repurposed to provide this support? A successful example is the reorientation of agricultural subsidies to support biodiversity conservation - could that be extended?*

**Author Response:** Thank you for guiding our attention to this issue. We referred to a report proving that less is more and that small-scale farming which relies on available resources is actually more profitable than large-scale ones with subsidies.

*Second is the question of equality of access. It is likely that food from small-scale production will be substantially more expensive (in a European context where labour is costly) than from large-scale. Foraging and community gardens are unlikely to be accessible to, say, residents of deprived urban areas. In any case, they would not have the time free for these activities. There is a significant literature on food inequality in urban areas that would be worth exploring.*

*Researchers have already noticed "the contemporary food sector...bifurcating into two main ‘zones’ of production: standardized, specialized production processes responding to economic standards of efficiency and competitiveness on the one hand; localized, specialized production processes attempting to trade on the basis of environmental, nutritional, or health qualities on the other." (Sonnino & Marsden). There seems a high risk that affluent consumers could enjoy the cash or time-expensive products advocated here, while leaving behind significant sectors of society.*

**Author Response:** Thank you for the raising this question! We evaluated this dimension but decided to exclude it from the manuscript as it would steer the discussion in an entirely different direction. We acknowledge the problem, but it is an issue outside the scope of the essay. The pillars we proposed are not a substitute for the current system per se (as there are currently no sustainable alternatives for producing cheap food that is affordable for everyone), but rather a safety net, a layer providing a short-term security buffer in times of need.

# Peer review: Reviewer response for version 1

**DOI:** 10.21956/openreseurope.14208.r26564

**Published:** 2021-04-08

**Authors:** Łukasz Łuczaj

**Affiliations:** 1 Institute of Biology and Biotechnology, University of Rzeszów, Rzeszów, Poland

The paper is a synthetic look at the possible ways of enhancing food security in Europe in the perspective of the current COVID pandemic. I think it is a valuable contribution to the discussion about it, as it takes into account culinary traditions and foraging as well as the issue of small scale producers. I agree with most ideas contained in the paper. However, the paper is quite brief, does not go into details and has too few references. It should be enriched with relevant references in all its sections, and give more specific examples. For example the authors say in the conclusions that we need “4) Drafting legislation allowing foraged/self-grown/home-prepared product exchange or sale in informal markets with less severe regulations compared with industrially produced food.” but do not give the example of current laws. Some countries already have such regulations (see Germany and Poland) but these regulations should be even more relaxed. I would also give example of Poland and France as the two countries which have a relatively large number of small farms, partly resisting the historical trend observed in places like Great Britain where agricultural land is owned by few large producers. As far as supporting small firms and farms is concerned, I recently discussed it in a blog post and proposed to look at the economy from the perspective of fractal geometry, underlining the importance of structural diversity of all agents of economic systems.

 Recently a number of papers and essay on foraging movement was published in Europe. It would enrich the article to quote them (and some references within):

- Łuczaj, Ł., Wilde, M. and Townsend, L., 2021. The Ethnobiology of Contemporary British Foragers: Foods They Teach, Their Sources of Inspiration and Impact ^1^
- Townsend, L.. 2020. The professional wild food community and Covid-19: The use of online platforms in supporting people to access alternative food sources ^2^
- Landor-Yamagata, J.; Kowarik, I.; Fischer, L. 2018. Urban foraging in Berlin: People, plants and practices within the metropolitan green infrastructure ^3^
- Luczaj L. 2020. On the Wild Side: From the Anthropology of Hunter-Gatherers to Postmodern Foraging, Bushcraft and New-Age Nature Seekers ^4^.

The allotment movement popular both within the ex-Communist and western Europe must be mentioned, e.g.

- Klepacki, P. and Kujawska, M., 2018. Urban allotment gardens in Poland: Implications for botanical and landscape diversity ^5^. This paper is a report from a project done in 2009-2012 by the Ethnographic Museum in Kraków documenting the importance of allotments in Poland, both in a cultural and biological context.
- Sowińska-Świerkosz, B., Michalik-Śnieżek, M. and Bieske-Matejak, A., 2021. Can Allotment Gardens (AGs) Be Considered an Example of Nature-Based Solutions (NBS) Based on the Use of Historical Green Infrastructure? ^6^

The expressions ‘organic farming’ and ‘permaculture’ are not mentioned in the paper, and they are important part of the food security discussion.

Is the argument persuasive and supported by appropriate evidence?

Partly

Is the work clearly and cogently presented?

Yes

Is the topic of the essay discussed accurately in the context of the current literature?

No

Does the essay contribute to the cultural, historical, social understanding of the field?

Yes

Reviewer Expertise:

Foraging, traditional food gathering, wildlife gardening, rewilding.

I confirm that I have read this submission and believe that I have an appropriate level of expertise to confirm that it is of an acceptable scientific standard, however I have significant reservations, as outlined above.

## References

[1] : The Ethnobiology of Contemporary British Foragers: Foods They Teach, Their Sources of Inspiration and Impact. *Sustainability* .2021;13(6) : 10.3390/su13063478 10.3390/su13063478

[2] : The professional wild food community and Covid-19: The use of online platforms in supporting people to access alternative food sources. *Local Development & Society* .2020;1(2) : 10.1080/26883597.2020.1797441 160-165 10.1080/26883597.2020.1797441

[3] : Urban Foraging in Berlin: People, Plants and Practices within the Metropolitan Green Infrastructure. *Sustainability* .2018;10(6) : 10.3390/su10061873 10.3390/su10061873

[4] : On the Wild Side: From the Anthropology of Hunter-Gatherers to Postmodern Foraging, Bushcraft and New-Age Nature Seekers.2020;

[5] : Urban Allotment Gardens in Poland: Implications for Botanical and Landscape Diversity. *Journal of Ethnobiology* .2018;38(1) : 10.2993/0278-0771-38.1.123 10.2993/0278-0771-38.1.123

[6] : Can Allotment Gardens (AGs) Be Considered an Example of Nature-Based Solutions (NBS) Based on the Use of Historical Green Infrastructure?. *Sustainability* .2021;13(2) : 10.3390/su13020835 10.3390/su13020835

# Response

**Published:** 2021-10-16

**Authors:** Raivo Kalle

**Affiliations:** University of Gastronomic Sciences, Italy

*The paper is a synthetic look at the possible ways of enhancing food security in Europe in the perspective of the current COVID pandemic. I think it is a valuable contribution to the discussion about it, as it takes into account culinary traditions and foraging as well as the issue of small scale producers. I agree with most ideas contained in the paper. However, the paper is quite brief, does not go into details and has too few references. It should be enriched with relevant references in all its sections, and give more specific examples.*

**Author Response:** The briefness of the paper was intentional; we really would not like to expand it too much so as not to lose focus of the message. It is just an essay, not a full research article.

*For example the authors say in the conclusions that we need “4) Drafting legislation allowing foraged/self-grown/home-prepared product exchange or sale in informal markets with less severe regulations compared with industrially produced food.” but do not give the example of current laws. Some countries already have such regulations (see Germany and Poland) but these regulations should be even more relaxed.*

**Author Response:** The sentence was rephrased to clarify that it refers to the easier inclusion of products and productions in the market.

*I would also give example of Poland and France as the two countries which have a relatively large number of small farms, partly resisting the historical trend observed in places like Great Britain where agricultural land is owned by few large producers. As far as supporting small firms and farms is concerned, I recently discussed it in a blog post and proposed to look at the economy from the perspective of fractal geometry, underlining the importance of structural diversity of all agents of economic systems.*

**Author Response:** Many thanks for the examples! We completely agree with the reviewer. With our opinion article, we want to say this in one way or another. Yes, in southern Poland, due to historical features, small farms have survived, but elsewhere in the country, production is also concentrated in the hands of large producers. As it turns out, Polish small farms can also no longer remain competitive in the market because the current subsidy per hectare is equal for both large and small farms, and European quality control standards create inequalities (see more: Wetzels, H. 2020.  Poland: small farms, big farms & EU relations. Agricultural and Rural Convention – ARC2020. 10. February. https://www.arc2020.eu/cap-poland-small-farms-big-farms-eu-relations/). This is also the focus of the first point of our argument outlined in the conclusions. In addition to France and Poland, a considerable number of small farms have survived in both Estonia and Italy. We did not specify a list of countries as the problems are the same regardless of the country and the solution would be to make the current system more flexible.

 We read the reviewer's blog with great interest [http://thewildfood.org/2020/03/should-we-make-world-economy-more-like-a-hologram-or-a-fractal/]. We agree with the reviewer's ideas. Nevertheless, this pandemic showed the vulnerability of existing global systems. Community-based and small supply chain systems are more flexible. However, right now the global system is affected by non-agricultural factors, such as the lack of hundreds of thousands of truck drivers across Europe and the unavailability of sea shipping containers. Waltner-Toews *et al* (2020) stated: “This pandemic offers society an occasion to open a fresh discussion on whether we now need to learn how to do science in a different way. Conscientious scientists and engaged citizens cannot allow this opportunity to pass.” [Waltner-Toews, D., Biggeri, A., De Marchi, B., Funtowicz, S., Giampietro, M., O'Connor, M., Ravetz, J.R., Saltelli, A., van der Sluijs, J. P. (2020). Pandemie post-normali. Perché CoViD-19 richiede un nuovo approccio alla scienza. Recenti progressi in medicina, 111(4), 202-204. In English: https://steps-centre.org/blog/postnormal-pandemics-why-covid-19-requires-a-new-approach-to-science/ ]. This is the reason why we highlighted six interconnected pillars. We also believe that the solution is to try to look at the objectives holistically, through a flexible approach. This would help to better increase society's social confidence and to achieve greater goals. 

*Recently a number of papers and essay on foraging movement was published in Europe. It would enrich the article to quote them.*

**Author Response: **Thank you for the suggested references; we incorporated some of them into the manuscript. The allotment movement popular both within the ex-Communist and western Europe must be mentioned, e.g. Klepacki, P. and Kujawska, M., 2018. Urban allotment gardens in Poland: Implications for botanical and landscape diversity. This paper is a report from a project done in 2009-2012 by the Ethnographic Museum in Kraków documenting the importance of allotments in Poland, both in a cultural and biological context. Sowińska-Świerkosz, B., Michalik-Śnieżek, M. and Bieske-Matejak, A., 2021. Can Allotment Gardens (AGs) Be Considered an Example of Nature-Based Solutions (NBS) Based on the Use of Historical Green Infrastructure? ( **https://doi.org/10.3390/su13020835** ) The expressions ‘organic farming’ and ‘permaculture’ are not mentioned in the paper, and they are important part of the food security discussion. Thank you for the suggested references; we have expanded a bit the discussion on this subject and incorporated them into the text.
